# Patterns in the dynamics of respiratory viruses after the COVID-19 pandemic: an observational, retrospective study using sentinel surveillance network data, Catalonia, 2022 to 2025

**DOI:** 10.2807/1560-7917.ES.2026.31.38.2600065

**Published:** 2026-09-24

**Authors:** Aida Perramon-Malavez, Ermengol Coma, Leonardo Mendez-Boo, Eduardo Hermosilla, Luca Basile, Francesc Fina, Andrés Antón, Jacobo Mendioroz, SIVIC Network Steering Group, SIVIC Network Steering Group, Francisca Aguado, Josefina Ayats, Cristina Boté, Esteve Fernández, Maria Nieves Larrosa, Maria Antonia Llopis, Xavier Martínez-Gómez, Montserrat Martínez-Marcos, Gemma Ricós-Furio, Sara Rodoreda, Beatriz Rosón-Hernández, Ariadna Sanz Escartin

**Affiliations:** 1 Primary Care Services Information System (SISAP), Institut Catala de la Salut (ICS), Barcelona, Spain; 2 SISAP Research Group (GR-SISAP), Fundació Institut Universitari per a la Recerca a l'Atenció Primària de Salut Jordi Gol I Gurina (IDIAP Jordi Gol), Barcelona, Spain; 3 Department of Health, Sub-Directorate General of Surveillance and Response to Public Health Emergencies, Public Health Secretariat, Government of Catalonia, Barcelona, Spain; 4 Respiratory Virus Unit, Virology Section, Microbiology Department, Vall d'Hebron Hospital Universitari, Vall d'Hebron Institut de Recerca (VHIR), Vall d'Hebron Barcelona Hospital Campus, Barcelona, Spain; 5 The members of the group are listed under collaborators

**Keywords:** dynamic, patterns, respiratory viruses, COVID-19, post-pandemic, surveillance

## Abstract

**BACKGROUND:**

Respiratory viruses are a major cause of morbidity and mortality worldwide. Their circulation patterns were markedly altered by the COVID-19 pandemic. The Information System for Infection Surveillance in Catalonia (SIVIC), established in 2022, provides continuous, population-representative sentinel surveillance integrating syndromic and microbiological data.

**AIM:**

Our aim was to describe the timing, regularity and overlap of epidemics across 18 respiratory viruses using microbiologically confirmed data from SIVIC.

**METHODS:**

We conducted an observational, retrospective study using weekly data from the SIVIC sentinel network in Catalonia between October 2022 and September 2025. We calculated weekly positivity for each virus and defined epidemic start, peak and end based on week-to-week positivity growth and decline. Mean timing and variability across epidemiological years were estimated after applying a 3-week moving average to compensate for fluctuations.

**RESULTS:**

Among 32,782 analysed samples, 21,278 (64.9%) tested positive for at least one virus. Distinct and recurrent epidemic patterns were observed. Respiratory syncytial virus and influenza viruses exhibited consistent sequential peaks in late autumn and early winter, while rhinovirus and enterovirus showed bimodal peaks in autumn and spring. Some viruses (human adenovirus, human parainfluenza 1 and 4, human coronavirus NL63) circulated year-round at low levels. In contrast, SARS-CoV-2 showed a persistent summer epidemic pattern.

**CONCLUSION:**

Most respiratory viruses in Catalonia have re-established regular seasonal dynamics, with SARS-CoV-2 displaying a distinct summer peak. Year-round sentinel surveillance for timely monitoring of viral circulation can help anticipate the timing and sequence of epidemics peaks.

**Key public health message**

**What did you want to address in this study and why?**

We wanted to understand when respiratory viruses (such as the flu, the common cold, or SARS-CoV-2) are more likely to spread in Catalonia. During the COVID-19 pandemic, the usual timing of these viruses changed, leading to a few years of unpredictable patterns. Five years later, we used a new monitoring system to find out what the current situation is and when each virus is expected to peak.

**What have we learnt from this study?**

We found that SARS-CoV-2 is, currently, not a winter virus. In recent years, it has shown clear peaks during the spring and summer, which is different from what was initially expected. We also observed that respiratory viruses mainly follow a predictable pattern, appearing one after another in a consistent order every year.

**What are the implications of your findings for public health?**

These results provide health authorities with a clearer map of which viruses are circulating at any given time. Knowing when each virus is likely to appear helps experts to decide when is the best time to launch vaccination campaigns or health advice, ensuring that protection is in place just before the viruses start to spread. It also helps the health system to prepare for periods of high demand.

## Introduction

Respiratory viruses are a central focus of global public health strategies, as they contribute substantially to morbidity and mortality across all age groups, particularly among young children and older adults [1,2]. Before the COVID-19 pandemic, many respiratory viruses exhibited well-characterised seasonal patterns, often peaking in winter months in temperate regions [3,4]. However, the emergence of SARS-CoV-2 and the widespread implementation of non-pharmaceutical interventions disrupted these established dynamics, suppressing the expected circulation of influenza viruses, respiratory syncytial virus (RSV) and other viruses, while rhinovirus in many settings appeared to be less supressed and to return to previous circulation sooner [5,6].

Post-pandemic resurgence studies have begun to document changes in timing, magnitude and synchronicity of respiratory virus epidemics globally [7]. In particular, analyses of multi-site surveillance data suggest that after a delayed re-emergence, many viruses gradually returned to pre-pandemic periodicities, albeit with notable shifts in relative order and temporal overlap [7]. Despite these advances, comprehensive quantification of overlap and temporal interspersion among a broad panel of respiratory viruses remains limited.

In Catalonia, a previous study [5] described those patterns until 2021, while the COVID-19 pandemic was ongoing. They found that influenza viruses virtually disappeared during 2020/21, while RSV became the dominant virus with a delayed peak in mid-June rather than during the expected period. Human adenovirus reappeared in early September, coinciding with the start of the school year, followed by human parainfluenza viruses from March 2021, which caused shorter but more intense outbreaks. Generally, rhinovirus circulated throughout the year, while RSV and influenza peaks occurred sequentially, RSV typically preceding influenza.

In June 2022, the Information System for Infection Surveillance in Catalonia (SIVIC) was established [8,9] to strengthen the surveillance of acute respiratory infections in the Catalan territory. The SIVIC introduced a year-round system combining syndromic and sentinel microbiological surveillance of respiratory viruses.

By leveraging microbiologically confirmed data from the SIVIC system, we aim to describe the timing and regularity of epidemics across a broad spectrum of 18 respiratory viruses, exploring how these epidemic periods overlap and interact.

## Methods

### Study design and settings

We conducted an observational, retrospective, cross-sectional study using weekly data from the sentinel microbiological surveillance system of the SIVIC [8] network in Catalonia, a temperate climate region in north-eastern Spain. These weekly data are based on the results of oro-nasopharyngeal swab samples collected from patients included in the SIVIC system who present with symptoms suggestive of acute respiratory infection (ARI) at participating sentinel primary care practices. This network comprises 33 such practices, covering 9% of the Catalan population and is representative in terms of sex, age, region and socioeconomic level [10]. Samples from symptomatic patients were collected following a systematic sampling protocol and sent to reference laboratories to be tested for viral infection [8]. Primary care professionals collect swabs from the first 15 patients each week per practice who meet the clinical criteria for ARI. Therefore, there are a maximum of 15 samples per week and practice. Each sample was tested with multiple reverse transcription PCR assays (RT-PCR) Allplex Respiratory Panel Panel Assays and Allplex SARS-CoV-2/FluA/FluB/RSV Assay (Seegene, South Korea) for the microbiological confirmation of up to 18 respiratory viruses [11]. The panel included human adenovirus (AdV), human bocavirus (BoV), human coronaviruses (CoV) 229E, NL63 and OC43, SARS-CoV-2, human enterovirus (EV), human metapneumovirus (MPV), human rhinovirus (RV), human influenza A virus (IAV; subtypes H1pdm09 and H3), human influenza B virus (IBV), human parainfluenza viruses 1 to 4 (PIV1–4), and human respiratory syncytial virus groups A and B (RSV-A, RSV-B). In cases where influenza A subtyping was inconclusive, the result was categorised as influenza A (unspecified).

### Immunisation context

Several immunisation campaigns against respiratory viruses were in place in Catalonia during the study period [8]. Vaccination campaigns for influenza and COVID-19 typically start in mid-September, targeting adults aged > 59 years and individuals < 60 years with underlying clinical risk factors. In addition, the influenza vaccine is offered to all children aged 6–59 months.

Regarding RSV, universal immunoprophylaxis with nirsevimab (a long-acting monoclonal antibody) is offered to all infants < 1 year of age facing their first RSV season, starting from October 2023. This campaign is organised into two cohorts: a *catch-up* cohort (infants born between April and September, immunised in primary care practices during October) and a *seasonal* cohort (infants born between October and March, immunised in the hospital shortly after birth) [12].

In the epidemiological year 2025/26, immunisation coverage for nirsevimab reached ca 93%. For influenza and COVID-19, coverage among individuals > 59 years was 48% and 36%, respectively, increasing to 68% and 55% in the age group > 79 years [8].

### Data collection

The study spanned October 2022 to September 2025, encompassing three complete epidemiological years. We obtained data from the SIVIC surveillance system that included information on week of sampling, total number of samples tested, virus detection results, sex and age of the patient, socioeconomic status and rurality.

Age groups were defined according to SIVIC classification: 0–11 months, 12–35 months, 3–4 years, 5–9 years, 10–14 years, 15–29 years, 30–44 years, 45–59 years, 60–69 years, 70–79 years and ≥ 80 years. Socioeconomic status was assessed using the MEDEA socioeconomic deprivation index stratifying urban practices in four groups [13]. Rural areas were defined as those with fewer than 10,000 inhabitants and a population density below 150 inhabitants per km^2^, in accordance with regional guidelines. Patients without demographic information (sex, age) were excluded.

### Statistical analyses

Continuous variables were summarised using mean and standard deviation (SD), while categorical variables are presented as frequencies and percentages.

Positivity rate was defined as the proportion of virus detections relative to the number of samples tested, expressed as percentage (%). Longitudinal trends in positivity were described using stacked bar charts by virus. Unspecific subtyping of Influenza A was excluded from this representation due to its low counts.

We also examined annual patterns, with an epidemiological year defined as October to September. Within each season, we measured start, peak and end of an epidemic for each studied virus that showed multiple epidemics throughout the study period. Given that there was no concrete quantitative definition of what constitutes an epidemic in the public health literature [14], we considered the epidemic start as the week after which the positivity for a virus grew for two consecutive weeks more than 20% of the previous week. Once start was defined, the positivity value that corresponded to the starting week was used as a threshold to define the end of the epidemic. If no value below that threshold was found, the minimum value after the epidemic peak was considered the end of the epidemic. We considered the week when the maximum value of positivity in an epidemiological year was reached as the epidemic peak week. Manual review and correction were conducted in cases of algorithmic limitations.

Illustrative plots showing the application of these definitions for each virus are provided in the Supplement. For greater clarity, we aggregated all types and subtypes of influenza and RSV under the respective labels 'influenza' (IV) and 'respiratory syncytial virus' (RSV) after confirmation of their observed low variability between the epidemiological years.

With the aim to detect and visualise patterns of viral circulation in Catalonia, we calculated the mean (SD) start, end and peak weeks per virus after applying a 3-week moving average to positivity for smoothing purposes. That way, we assessed consistency between epidemiological years within viruses and consecutiveness of the different viral epidemics. In addition, we provide in the Supplement the virus-to-virus delays in start, peak and end weeks of their epidemics.

## Results

From October 2022 to September 2025, 33,228 nasopharyngeal swab samples were collected from the SIVIC sentinel primary care practices centres. From those, we excluded 446 (1.3%) samples that were not linked to sociodemographic information. From the final 32,782 samples (people tested), 21,278 (64.9%) were positive to at least one of the 18 viruses tested. The Table shows the characteristics of patients included in the sentinel system and those with at least one positive sample. In Supplementary Table S1, these data can be seen stratified by epidemiological year.

**Table:** Population included in the sentinel microbiological surveillance system, Catalonia, Spain, October 2022–September 2025 (n = 32,782)

Variable	Total (n = 32,782)	Column %	Positive (n = 21,278)	Row %
Sex				
Female	18,907	57.7	12,180	64.4
Male	13,875	42.3	9,098	65.6
Age group				
0–11 months	545	1.7	445	81.7
12–35 months	932	2.8	779	83.6
3–4 years	549	1.7	422	76.9
5–9 years	843	2.6	556	66.0
10–14 years	875	2.7	550	62.9
15–29 years	5,585	17.0	3,693	66.1
30–44 years	7,355	22.4	4,740	64.4
45–59 years	7,468	22.8	4,815	64.5
60–69 years	3,780	11.5	2,311	61.1
70–79 years	3,065	9.3	1,902	62.1
≥ 80 years	1,785	5.4	1,065	59.7
Socioeconomic status^a ^				
R	10,883	33.2	7,039	64.7
1U	4,129	12.6	2,606	63.1
2U	2,924	8.9	1,923	65.8
3U	5,611	17.1	3,691	65.8
4U	9,235	28.2	6,019	65.2
Epidemiological year				
2022/23	13,149	40.1	8,745	66.5
2023/24	10,527	32.1	6,465	61.4
2024/25	9,106	27.8	6,068	66.6

^a^ Rural areas (R): non-urban settings. Urban areas (U): divided into four levels based on the socioeconomic deprivation index: 1U: urban with low deprivation (high socioeconomic status). 2U: urban with medium-low deprivation. 3U: urban with medium-high deprivation. 4U: urban with high or very high deprivation (vulnerable settings or low socioeconomic status).

Slightly more than half of the samples were from female patients (57.7%), with similar positivity between sexes. Positivity was highest (> 80%) among children < 2 years, with a progressive decrease with increasing age. Regarding socioeconomic status, a substantial proportion of samples were obtained from patients living in rural (33.2%) and urban most deprived areas (28.2%), where the positivity rate was similar to other groups. Overall positivity ranged from 61.4% to 66.5% during the 3-year period.

**Figure 1:**
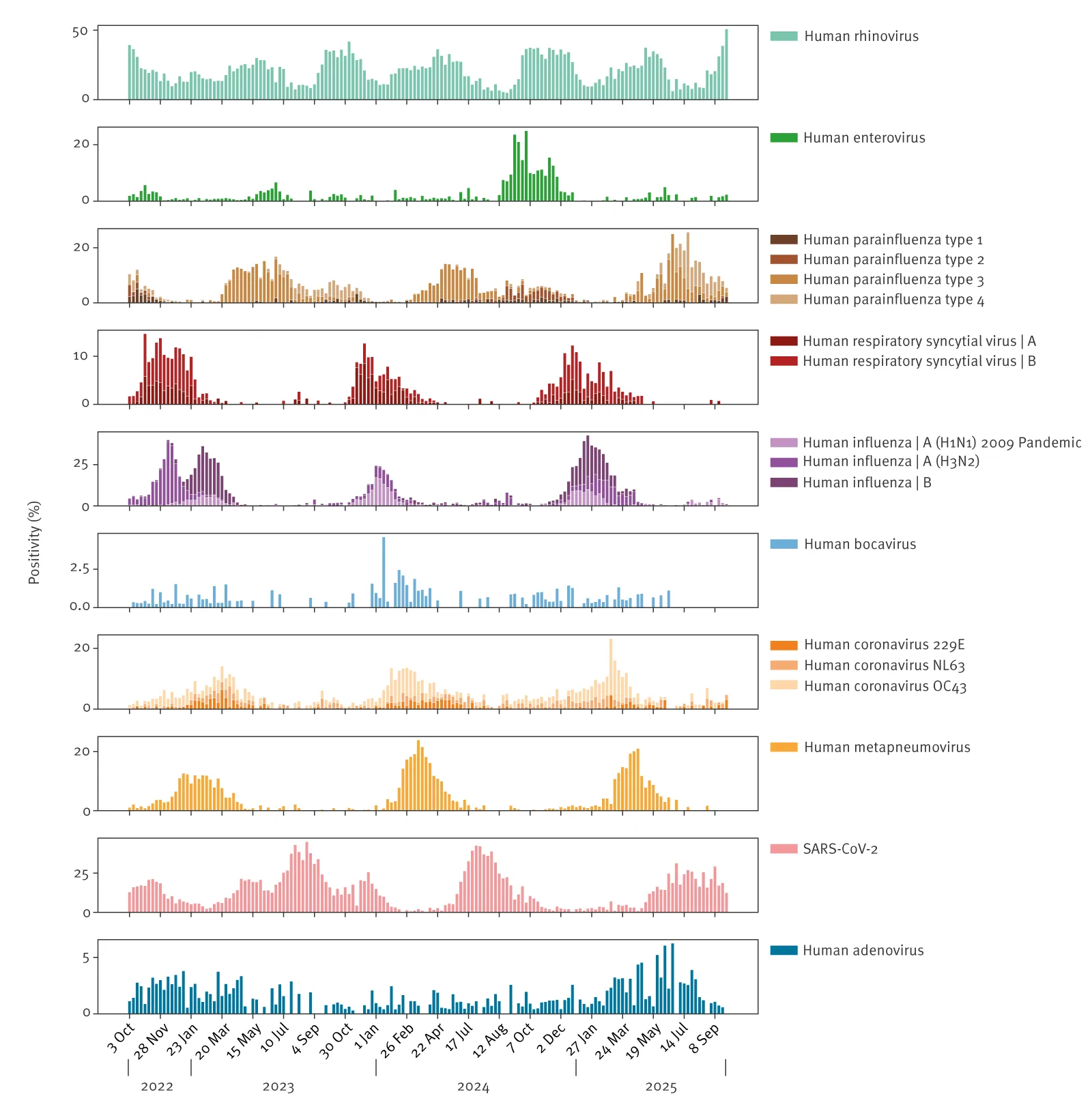
Stacked positivity values for 18 viruses detected by multiple RT-PCR, sentinel system, Catalonia, Spain, October 2022–September 2025 (n = 32,782 samples)

Overall positivity values for each virus are shown in Figure 1. Some viruses displayed a clear seasonal pattern, while others were detected at low levels throughout the study period. IAV showed a distinct annual epidemic pattern and IBV a less regular seasonality. The RSV epidemics occurred during the increasing phase of the influenza season, while PIV-2 appeared slightly earlier and MPV during the declining phase, followed by PIV-3. Infections with SARS-CoV-2 and RV were detected year-round, although RV positivity decreased during SARS-CoV-2 epidemic periods. Human enterovirus circulated year-round and demonstrated a dual epidemic pattern comparable to RV, while showing increased detection in August 2024. Also AdV, BoV and the seasonal CoVs were observed throughout the period with generally lower positivity rates.

**Figure 2:**
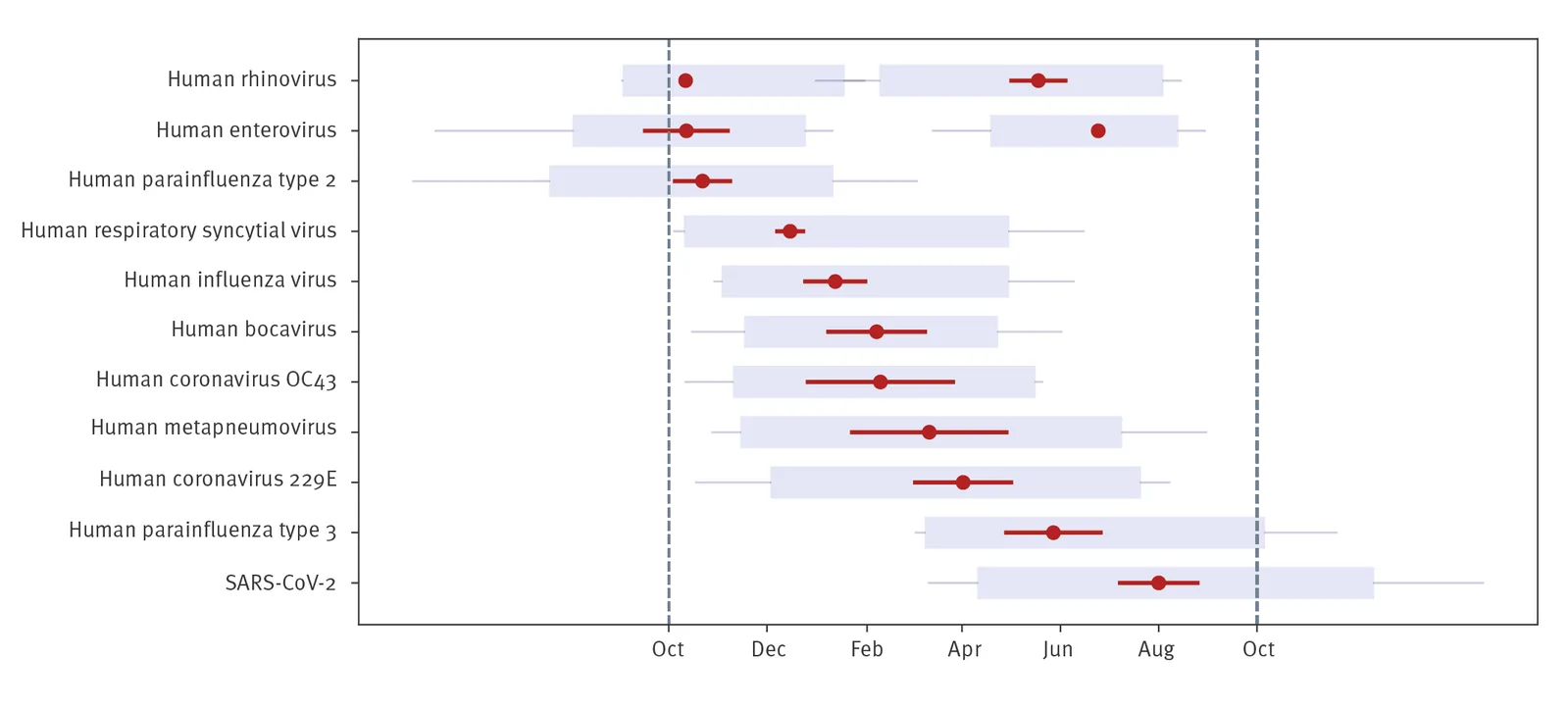
Viruses’ chronological occurrence, by week of first average epidemic peak since the start of the epidemiological year in October, aggregated data, Catalonia, Spain, 2023/24–2024/25 (n = 32,782)

All viruses but AdV, CoV-NL63, PIV-1 and PIV-4 have shown enough detections and stability to define epidemic outbreaks. The mean (SD) epidemic period and peak per virus presenting definable outbreaks are depicted in Figure 2 through a candlestick plot showing the average epidemic period of each studied virus in Catalonia’s viral landscape. For RV and EV, their dual epidemic pattern has been considered.

Light blue candlestick bars: mean start and end of each virus epidemic (SD shown as whiskers); red dots with error bars: epidemic peak. Both Influenza virus and RSV types are shown combined in the figure.

Both EV and RV exhibited nearly concurrent epidemic peaks at the start of the epidemiological year. For EV, we saw a broader and less defined ascending phase that overlapped with the PIV-2 epidemic, which reached its maximum shortly later. While EV and PIV-2 typically initiated circulation in spring, RV surged in September. Nevertheless, these three viruses all peaked between October and November.

The RSV activity began in October, followed by the onset of influenza virus circulation in early November. The RSV peak occurred approximately 1 month earlier than the influenza peak. While influenza epidemics displayed a symmetric pattern with a rapid decline, RSV maintained a longer descending phase, resulting in overlapping epidemic periods.

Circulation of seasonal CoV-OC43 and BoV began between October and November, with extended epidemic peaks between February and April. Their activity coincided with that of MPV, which reached its maximum slightly later, and with CoV-229E, which reached the highest positivity values around April.

Following the decline of CoV-OC43 and BoV, RV exhibited a second epidemic peak coinciding with PIV-3, followed by a second EV epidemic. Subsequently, SARS-CoV-2 exhibited a distinct epidemic peak between August and September, a period characterised by minimal activity of other respiratory viruses. Notably, although the predominant circulation of SARS-CoV-2 from 2023 onwards occurred during the summer months, a minor epidemic wave was still observed in winter 2022, preceding the influenza peak (Figure 1). A similar but less pronounced pattern was detected in winter 2023. However, this winter resurgence was no longer evident in 2024. Remarkably, the onset of the summer epidemic occurred at almost identical time points during the past two consecutive epidemiological years, suggesting the emergence of a stable seasonal pattern.

## Discussion

In this study, we provide a systematic description of the circulation dynamics of 18 respiratory viruses in a post-COVID-19 pandemic context. Using data from the well-established Catalan primary care sentinel microbiological surveillance system, we identified distinct viral temporal patterns and interactions, suggesting a re-emergence of regular epidemic cycles following the pandemic disruption. Some viruses, such as AdV, PIV types 1 and 4, and seasonal CoV-NL63, maintained low but steady activity across the year, while others displayed well-defined epidemic waves. Among these, the RSV and influenza viruses showed the most regular and marked seasonality, typically peaking consecutively between late autumn and early winter. IAV showed a distinct annual epidemic pattern, IBV presented a less regular seasonality, in agreement with previous literature [15,16]. The RV and EV were present throughout the year, but their circulation intensified in autumn and spring, resulting in a bimodal epidemic pattern and potentially filling inter-epidemic windows between the activity of other viruses.

Even though seasonality in a pandemic context is a complex phenomenon modulated mainly by the population's susceptibility to infection, transitory immunity and viral evolution, some early pandemic studies anticipated that SARS-CoV-2 would eventually adopt a winter epidemic pattern like influenza viruses [17]. This was supported, in Catalonia, by the massive Omicron wave in December 2021 and January 2022 [18]. Nevertheless, our 3-year post-pandemic analysis revealed a different trend. In Catalonia, recent SARS-CoV-2 epidemics typically began in spring and reached their peak between August and September, preceding a decline that extended into early winter. Similar summer peaks have recently been reported in the United Kingdom [19], contrasting with early pandemic hypotheses that emphasised winter transmission and an influenza-like pattern, which were often based on environmental and behavioural factors alone [19,20]. Ongoing surveillance and comparative analyses need to confirm whether this new seasonal profile represents a stable pattern, or it is still a transitional phase. If it proves to be stable, a reassessment of current vaccination strategies, including the timing and scope of existing campaigns and the potential utility of newly developed combined vaccines, may be warranted to ensure optimal alignment with viral circulation.

Our results extend previous studies describing the reconfiguration of respiratory virus seasonality after the COVID-19 pandemic. Observations from southern China and Mexico also reported asynchronous and prolonged viral activity following the relaxation of control measures [6,21], which is in line with our findings that also revealed a later gradual re-establishment of epidemic periodicity, yet with altered timing and overlap. Also, an extensive analysis of data from Cleveland, United States, reported results consistent to ours regarding AdV, EV and RV annual circulation, the latter showing semi-annual cycles [22]. However, our results diverged from other descriptions. A review published in 2020 [23] defined the epidemic period of RSV as starting in November, whereas our data showed an earlier onset. Moreover, while that review described BoV as a year-round virus and EV as predominantly summer-circulating, our observations indicate a clear epidemic pattern for BoV and a dual, bimodal profile for EV, with peaks in autumn and spring. These differences may reflect regional variations in climate, social behaviour and population structure, in addition to possible post-pandemic shifts in viral interactions and immunity.

Importantly, most comparable studies [21,24] describe qualitative seasonal shifts or quantify trends through interrupted time series, without formally characterising epidemic timing. A key contribution of our work is the systematic definition of mean epidemic onset, peak and end for each pathogen, providing an operational reference framework for public health planning that, to our knowledge, has not been previously established for this breadth of viruses in a Mediterranean temperate setting.

A previous Catalan study from 2024 that focused on fewer pathogens and earlier epidemiological years (from 2016 up to 2021), also identified delayed or broadened peaks for several viruses [5]. The present work expands these findings by including additional viruses and more recent data. Notably, while that earlier study described sharper PIV peaks, we observed broader, more protracted waves across epidemiological years, possibly reflecting ongoing adjustments in viral coexistence and host immunity. Nevertheless, whereas the present analysis is based on sentinel surveillance data, the Information plan for acute respiratory infections in Catalonia (PIDIRAC) data used in the earlier study lacked consistent and representative sampling across Catalonia, which could also explain the differences. On the other hand, new immunisation campaigns have been introduced in Catalonia (for instance, nirsevimab against RSV in October 2023) and may have added perturbations to the viral circulation patterns in comparison with previous years. Further surveillance during the next years is needed to confirm if there will be a definitive consolidation of its seasonal pattern.

Recent literature has also explored interactions between respiratory viruses and their possible ecological interference [7,25,26]. Reviews of respiratory virus seasonality [23] suggested that innate immunity-driven host competition, cross-immunity, particularly among phylogenetically related viruses, and environmental drivers (e.g. temperature and humidity) contribute to stagger epidemic timing. The observation in our study that several viruses exhibited non-overlapping peaks supports these hypotheses of exclusive susceptibility and temporary transversal immunity [25].

Nonetheless, certain limitations must be acknowledged. The SIVIC sentinel network was established after the onset of the COVID-19 pandemic, preventing comparisons with pre-pandemic or pandemic periods. Only three epidemiological years of surveillance were available, which may limit our ability to distinguish short-term fluctuations from long-term shifts in viral dynamics. In addition, a different definition of epidemic season might alter our results. Besides, as the system was not designed for detailed age-specific analyses, some subgroup-specific differences could not be assessed. Moreover, variability in healthcare-seeking behaviour may have influenced the observed detection frequencies [23].

Despite these limitations, the systematic and representative design of the sentinel system for respiratory virus surveillance, its integration within primary care, and the use of standardised protocols across laboratories provide robust and consistent data for temporal trend analysis. The use of standardised molecular diagnostics (RT-PCR) guarantees high analytical sensitivity and comparability across viruses and epidemiological years [27,28]. In addition, this extensive post-pandemic analysis encompasses 18 respiratory viruses across three complete epidemiological years (2022/25), including more than 30,000 samples. The sentinel system's design,  which incorporates primary care teams based on their region, population type and socioeconomic level, ensures broad population representativeness, capturing both urban and rural areas and all age groups [29]. Moreover, continuous year-round sampling allows the precise delineation of epidemic onsets, peaks and overlaps, an advantage rarely achieved in traditional seasonal surveillance. Finally, the systematic characterisation of mean epidemic onset, peak and end for each pathogen provides public health authorities with a practical reference framework to anticipate epidemic waves, strengthen healthcare system preparedness during periods of high demand, improve clinical diagnosis by informing clinicians of which viruses are likely to be circulating at any given time, and optimise the timing of vaccination campaigns.

## Conclusion

The shift of SARS-CoV-2 towards a summer, seasonal epidemic, challenges previous assumptions of its winter predominance and raises questions regarding the timing and effectiveness of current public health campaigns in temperate regions. This study shows the importance of maintaining robust and year-round sentinel surveillance systems capable of monitoring multiple respiratory pathogens simultaneously and detecting changes in their epidemiological behaviour. Further studies, ideally encompassing longer temporal series and diverse regions, are warranted to confirm these observations. Adapting public health strategies to the evolving epidemiology and nature of respiratory viruses is key to optimise prevention and control measures in the post-pandemic era.

## Data Availability

Publicly available datasets were analysed in this study. These data can be found here: https://sivic.salut.gencat.cat/dades_obertes.

## Supplementary Data

2183000 Supplement
