# Supplementary material for: Patterns in the dynamics of respiratory viruses after the COVID-19 pandemic: an observational, retrospective study using sentinel surveillance network data, Catalonia, 2022 to 2025
Source: Euro Surveill. 2026 Sep 24;31(38):2600065. doi: 10.2807/1560-7917.ES.2026.31.38.2600065 (PMC13615385; doi:10.2807/1560-7917.ES.2026.31.38.2600065)

This supplementary material is hosted by Eurosurveillance as supporting information alongside the article "Patterns in the dynamics of respiratory viruses in Catalonia in the post-pandemic era: an observational, retrospective study using sentinel surveillance network data", on behalf of the authors, who remain responsible for the accuracy and appropriateness of the content. The same standards for ethics, copyright, attributions and permissions as for the article apply. Supplements are not edited by Eurosurveillance and the journal is not responsible for the maintenance of any links or email addresses provided therein.

Supplementary Material

For the manuscript "Patterns in the dynamics of respiratory viruses in Catalonia in the post-pandemic era: an observational, retrospective study using sentinel surveillance network data".

List of Contents

Supplementary Tables...2
Supplementary Figures 5

Supplementary Tables

Table S1. Descriptive table of the population included within the sentinel microbiological surveillance system of Catalonia, from October 2022 to September 2025 (n=32,782). Stratified by season from 2022-2023 to 2024-2025.

Variable		Total | 2022-2023 (N = 13,149)	%	Positive | 2022-2023 (N = 8,745)	%	Total | 2023-2024 (N = 10,527)	%	Positive | 2023-2024 (N = 6,465)	%	Total | 2024-2025 (N = 9,106)	%	Positive | 2024-2025 (N = 6,068)	%
Sex	Women	7,667	58.3	5,027	65.6	6,081	57.8	3,702	60.9	5,159	56.7	3,451	66.9
	Men	5,482	41.7	3,718	67.8	4,446	42.2	2,763	62.1	3,947	43.3	2,617	66.3
Age group	0-11 months	224	1.7	180	80.4	198	1.9	166	83.8	123	1.4	99	80.5
	12-23 months	410	3.1	340	82.9	258	2.5	213	82.6	264	2.9	226	85.6
	3-4 years	265	2.0	211	79.6	167	1.6	122	73.1	117	1.3	89	76.1
	5-9 years	357	2.7	256	71.7	260	2.5	149	57.3	226	2.5	151	66.8
	10-14 years	381	2.9	271	71.1	282	2.7	134	47.5	212	2.3	145	68.4
	15-29 years	2,183	16.6	1,473	67.5	1,781	16.9	1,098	61.7	1,621	17.8	1,122	69.2
	30-44 years	2,841	21.6	1,918	67.5	2,383	22.6	1,414	59.3	2,131	23.4	1,408	66.1
	45-59 years	2,884	21.9	1,900	65.9	2,455	23.3	1,503	61.2	2,129	23.4	1,412	66.3
	60-69 years	1,549	11.8	940	60.7	1,234	11.7	739	59.9	997	10.9	632	63.4
	70-79 years	1,323	10.1	806	60.9	946	9.0	585	61.8	796	8.7	511	64.2

	**≥80 years**	732	5.6	450	61.5	563	5.3	342	60.7	490	5.4	273	55.7
**Socioeconomic status**	**R**	4,304	32.7	2,874	66.8	3,626	34.4	2,182	60.2	2,953	32.4	1,983	67.2
	**1U**	1,656	12.6	1,067	64.4	1,404	13.3	850	60.5	1,069	11.7	689	64.5
	**2U**	1,505	11.4	1,003	66.6	829	7.9	538	64.9	590	6.5	382	64.7
	**3U**	2,234	17.0	1,511	67.6	1,745	16.6	1,074	61.5	1,632	17.9	1,106	67.8
	**4U**	3,450	26.2	2,290	66.4	2,923	27.8	1,821	62.3	2,862	31.4	1,908	66.7

Table S2. Definition of epidemic period per virus and season of study. Note that not all viruses present the same number of seasons due to their different epidemic periods.

Virus	Season	Start (week)	Peak (week)	End (week)	Growth duration (weeks)	Decrease duration (weeks)	Epidemic duration (weeks)
BV	2022-2023	10/10/2022	2/1/2023	29/5/2023	12	21	33
	2023-2024	4/12/2023	26/2/2024	29/4/2024	12	9	21
	2024-2025	7/10/2024	24/2/2025	7/7/2025	20	19	39
CoV-229E	2022-2023	10/10/2022	20/3/2023	24/7/2023	23	18	41
	2023-2024	1/1/2024	6/5/2024	5/8/2024	18	13	31
	2024-2025	30/12/2024	10/3/2025	30/6/2025	10	16	26
CoV-OC43	2022-2023	21/11/2022	19/12/2022	22/5/2023	4	22	26
	2023-2024	4/12/2023	26/2/2024	13/5/2024	12	11	23
	2024-2025	7/10/2024	17/3/2025	12/5/2025	23	8	31
IV	2022-2023	7/11/2022	26/12/2022	10/4/2023	7	15	22
	2023-2024	6/11/2023	8/1/2024	1/4/2024	9	12	21
	2024-2025	28/10/2024	3/2/2025	16/6/2025	14	19	33
IV-A	2022-2023	7/11/2022	19/12/2022	27/3/2023	6	14	20
	2023-2024	6/11/2023	8/1/2024	25/3/2024	9	11	20
	2024-2025	21/10/2024	3/2/2025	23/6/2025	15	20	35
IV-B	2022-2023	17/10/2022	20/2/2023	15/5/2023	18	12	30
	2023-2024	20/11/2023	18/3/2024	24/6/2024	17	14	31
	2024-2025	4/11/2024	27/1/2025	5/5/2025	12	14	26
MpV	2022-2023	14/11/2022	16/1/2023	8/5/2023	9	16	25
	2023-2024	4/12/2023	25/3/2024	5/8/2024	16	19	35
	2024-2025	28/10/2024	21/4/2025	11/8/2025	25	16	41
SARS-CoV-2	2022-2023	6/3/2023	28/8/2023	12/2/2024	25	24	49
	2023-2024	29/4/2024	8/7/2024	23/12/2024	10	24	34
	2024-2025	28/4/2025	28/7/2025	29/9/2025	13	9	22
RV	2022-2023	27/3/2023	5/6/2023	7/8/2023	10	9	19
	2023-2024	4/9/2023	9/10/2023	8/1/2024	5	13	18
	2023-2024	8/1/2024	29/4/2024	12/8/2024	16	15	31
	2024-2025	2/9/2024	14/10/2024	27/1/2025	6	15	21
	2024-2025	27/1/2025	19/5/2025	21/7/2025	16	9	25
RSV	2022-2023	3/10/2022	5/12/2022	6/3/2023	9	13	22
	2023-2024	16/10/2023	18/12/2023	27/5/2024	9	23	32
	2024-2025	14/10/2024	23/12/2024	26/5/2025	10	22	32
PiV-2	2023-2024	18/9/2023	9/10/2023	4/12/2023	3	8	11
	2024-2025	20/5/2024	4/11/2024	17/2/2025	24	15	39
PiV-3	2022-2023	6/3/2023	1/5/2023	27/11/2023	8	30	38
	2023-2024	4/3/2024	20/5/2024	9/9/2024	11	16	27
	2024-2025	17/3/2025	30/6/2025	8/9/2025	15	10	25
EV	2022-2023	3/10/2022	31/10/2022	12/12/2022	4	6	10
	2022-2023	15/5/2023	26/6/2023	31/7/2023	6	5	11
	2024-2025	3/6/2024	23/9/2024	6/1/2025	16	15	31
	2024-2025	24/3/2025	23/6/2025	25/8/2025	13	9	22

Supplementary Figures

Figure S1. Number of samples collected per week of the study period.

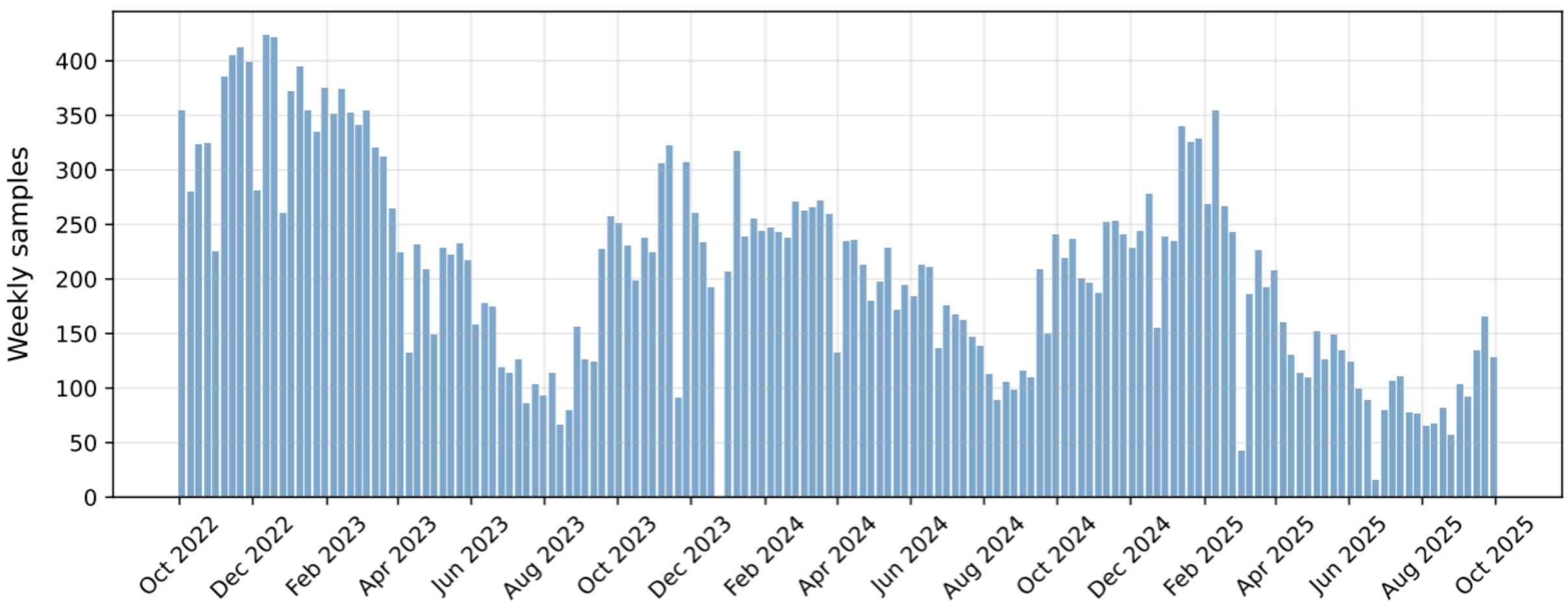

Figure S2. Definition of each epidemic of bocavirus.

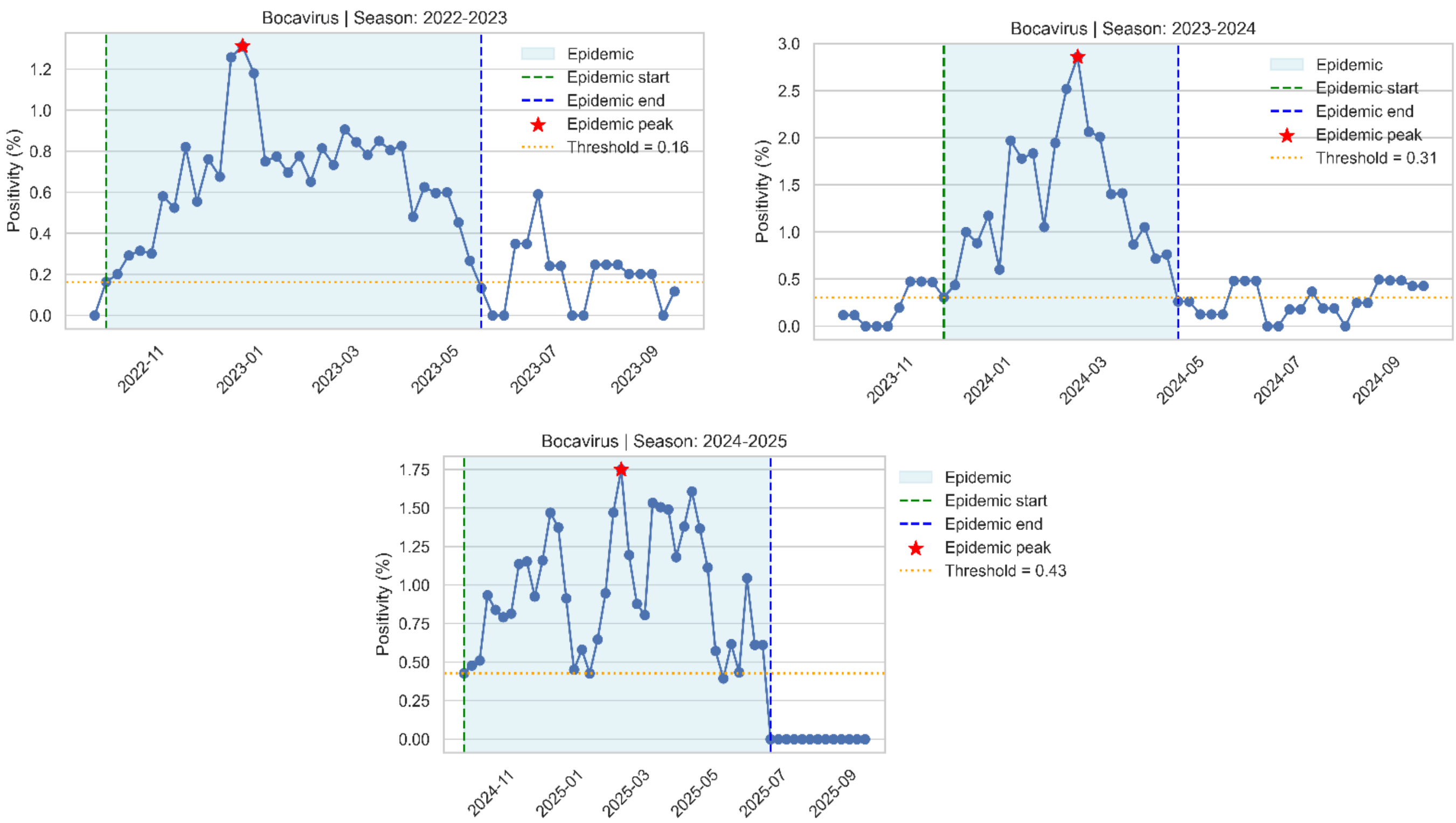

Figure S3. Definition of each epidemic of coronavirus 229E.

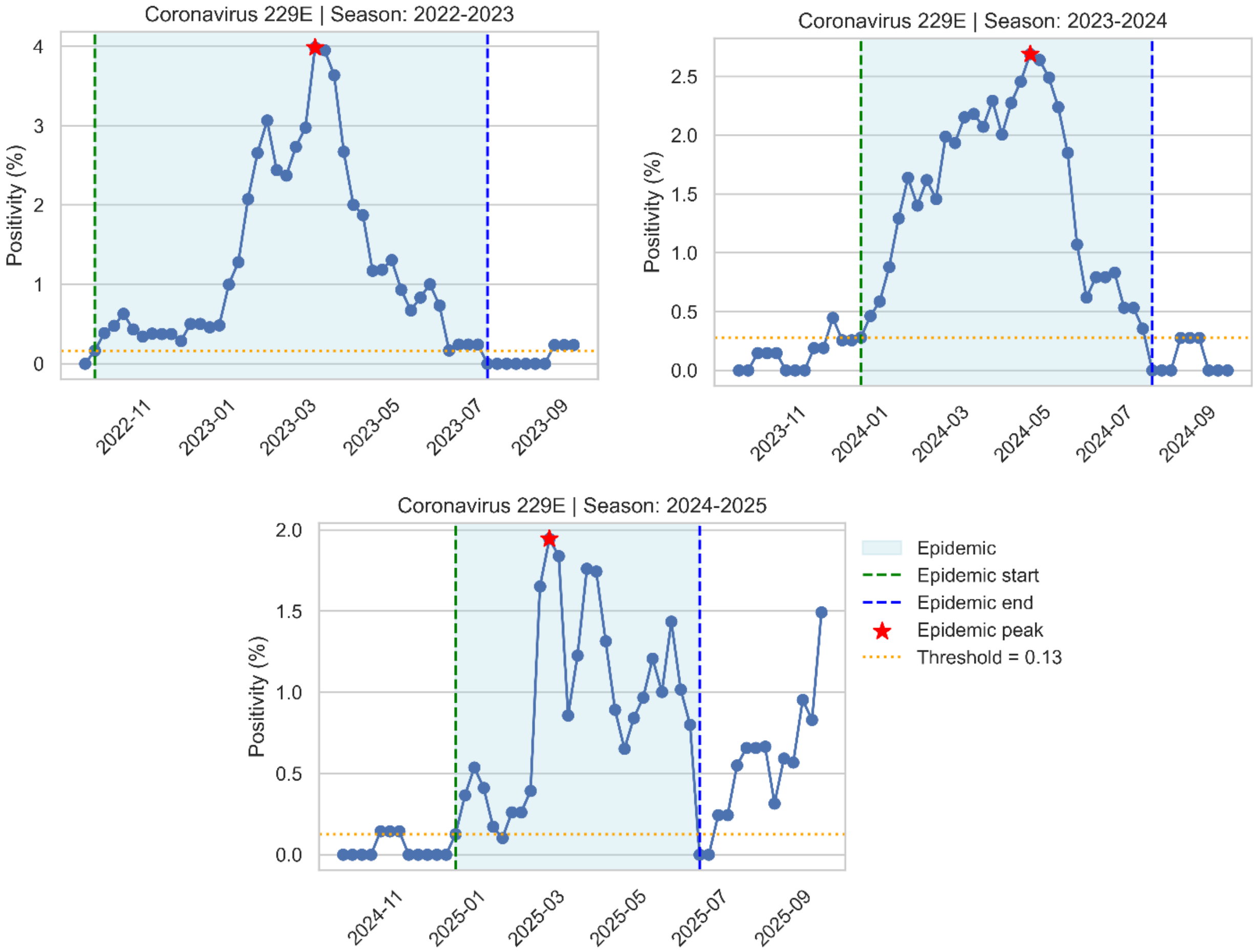

Figure S4. Definition of each epidemic of coronavirus OC43

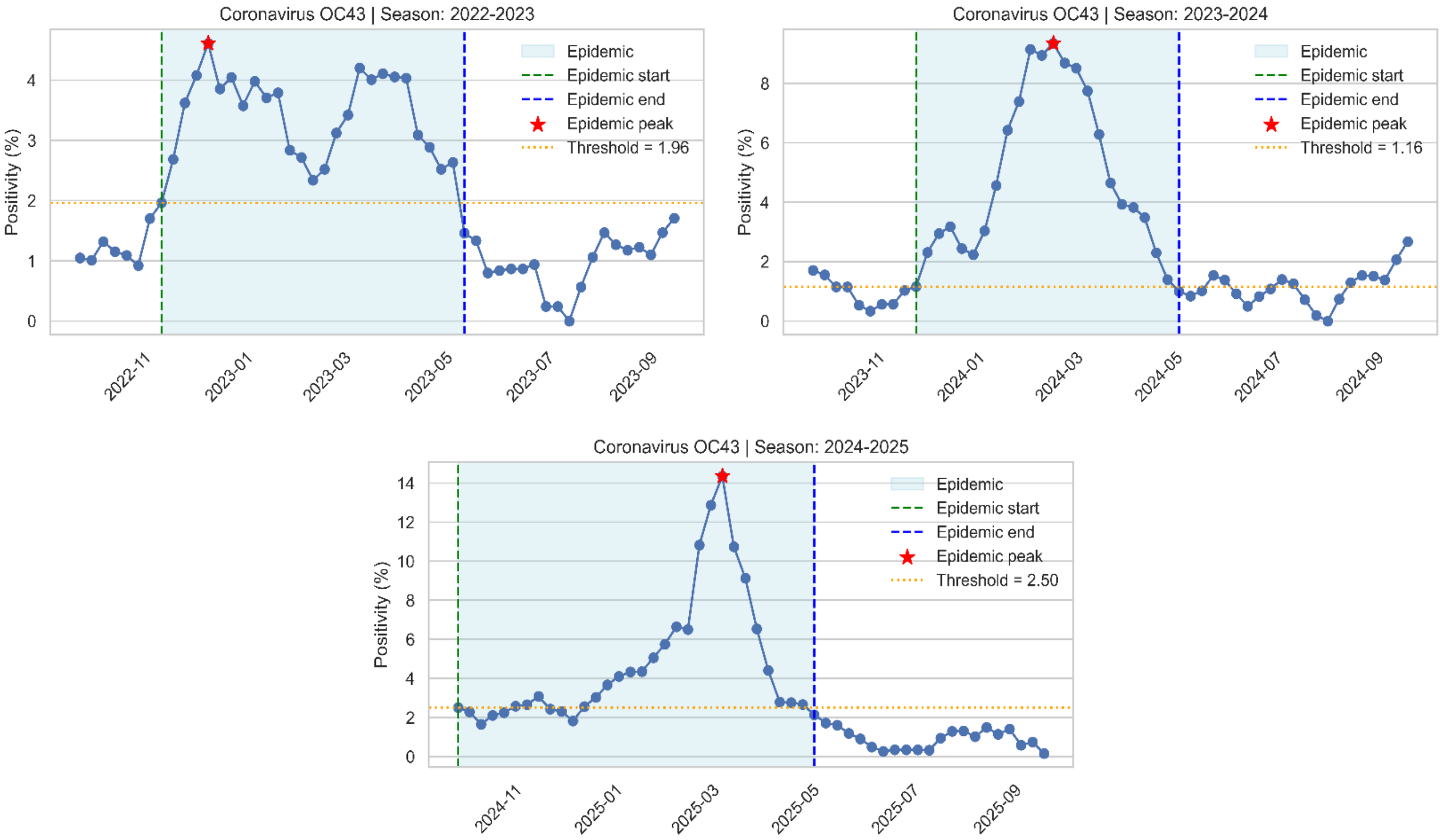

Figure S5. Definition of each epidemic of enterovirus.

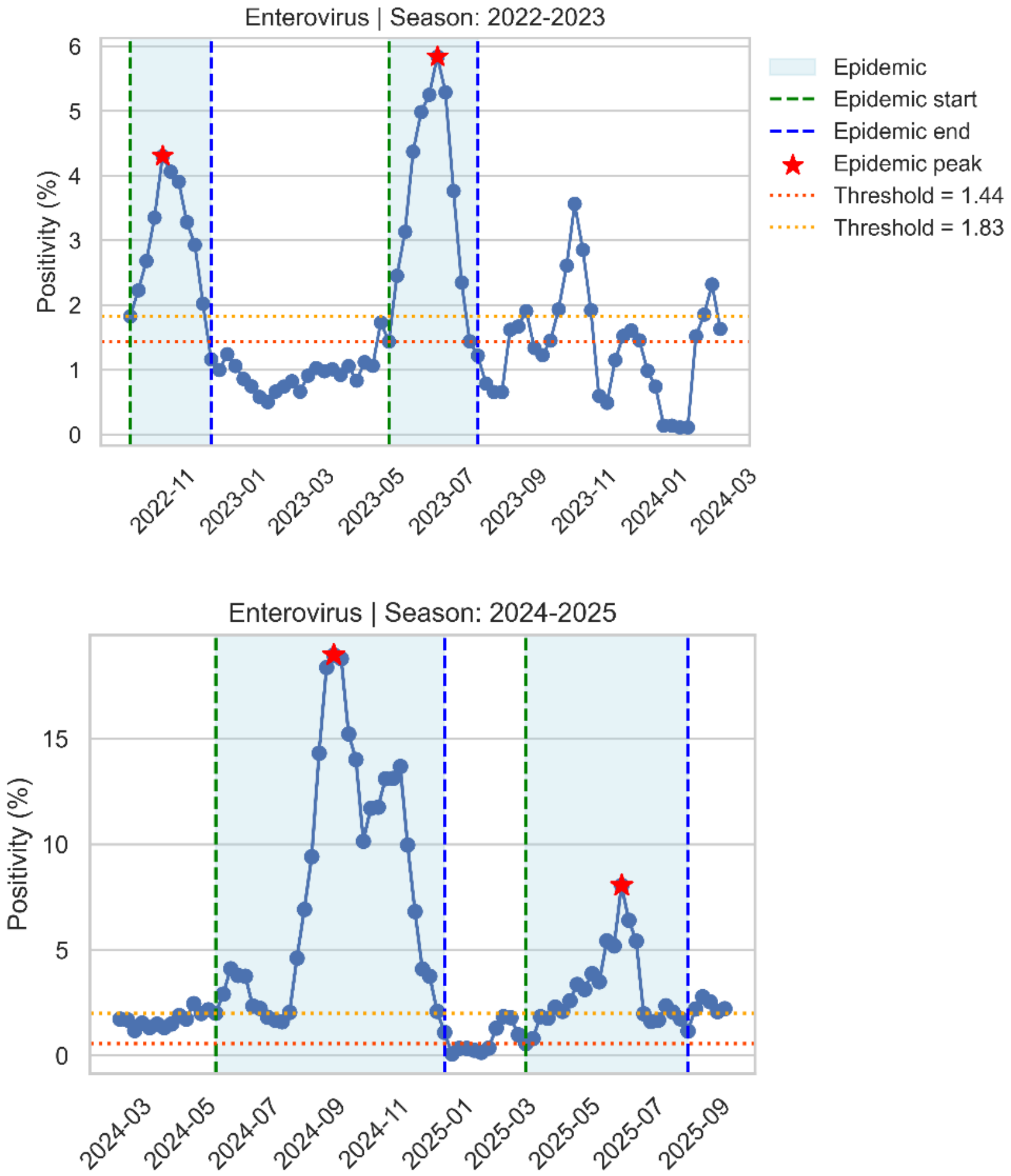

Figure S6. Definition of each epidemic of influenza.

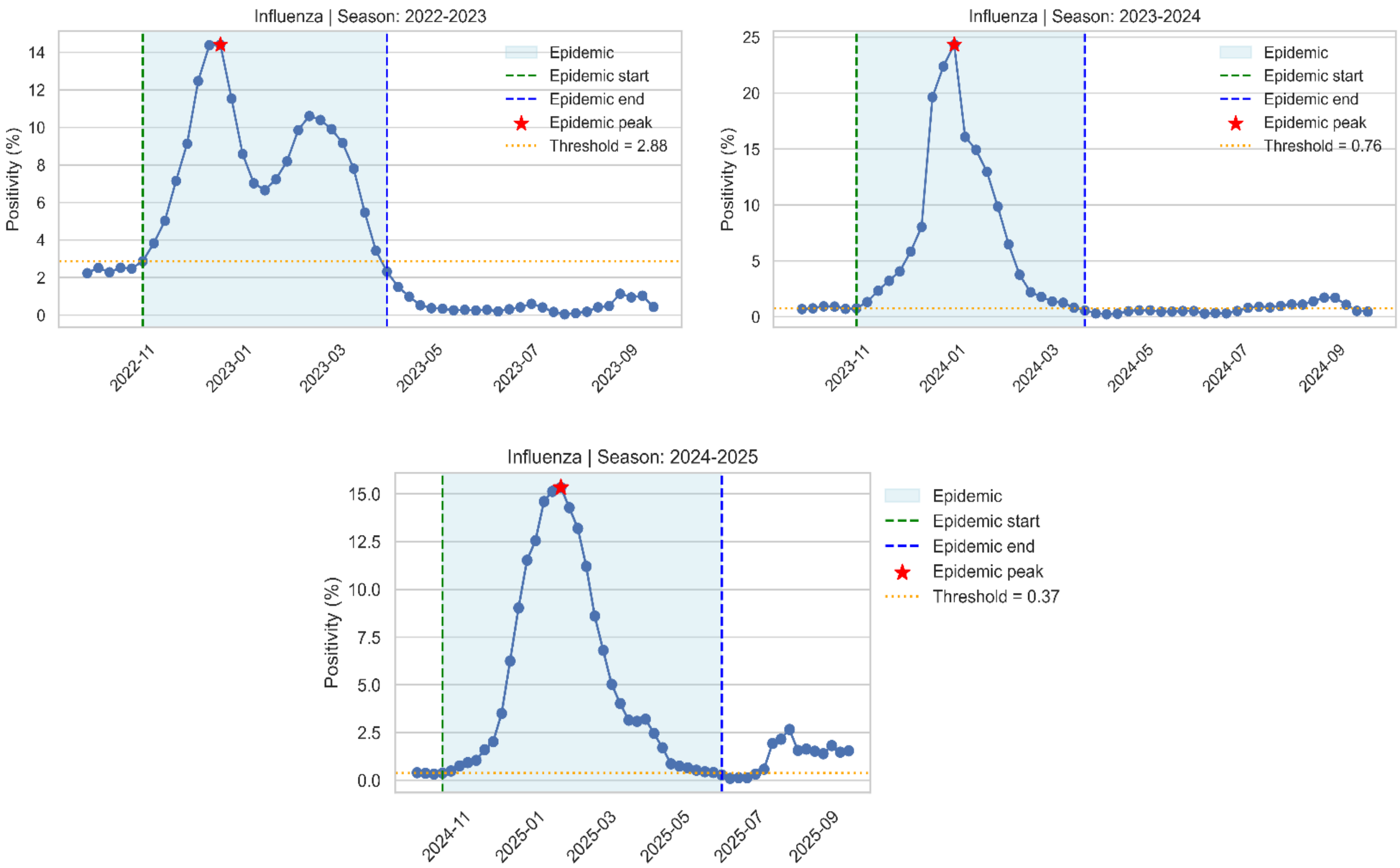

Figure S7. Definition of each epidemic of influenza A.

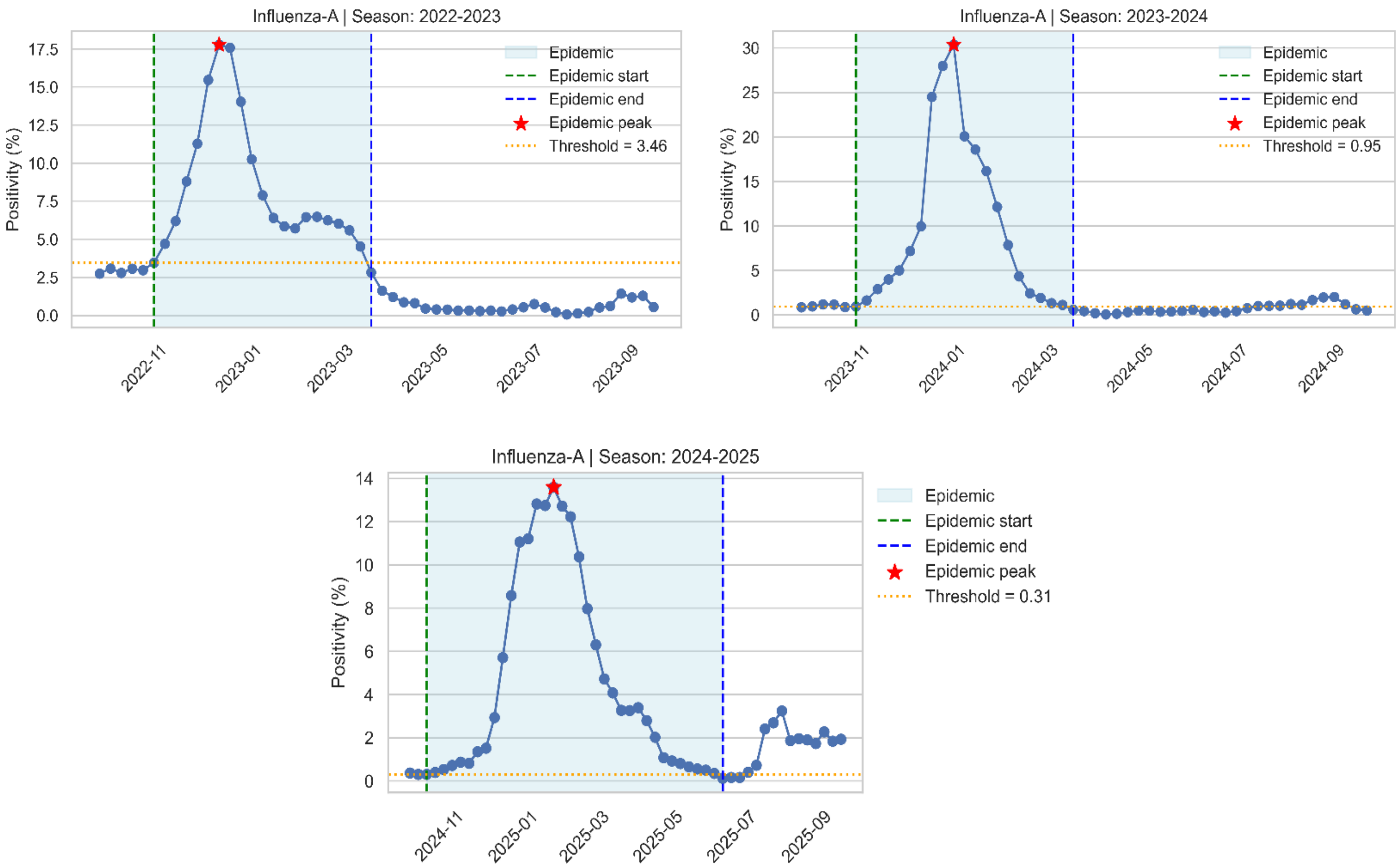

Figure S8. Definition of each epidemic of influenza B.

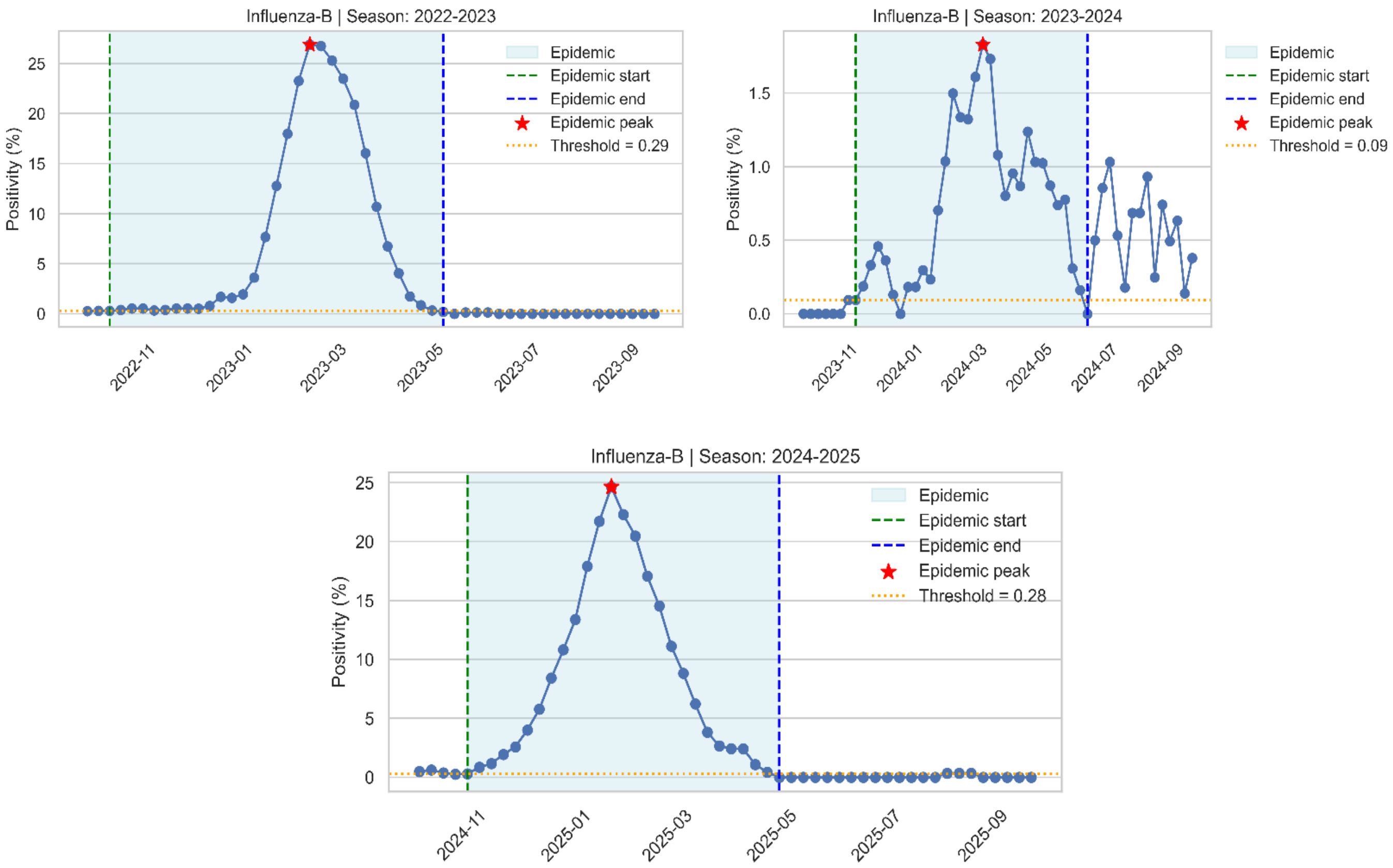

Figure S9. Definition of each epidemic of metapneumovirus.

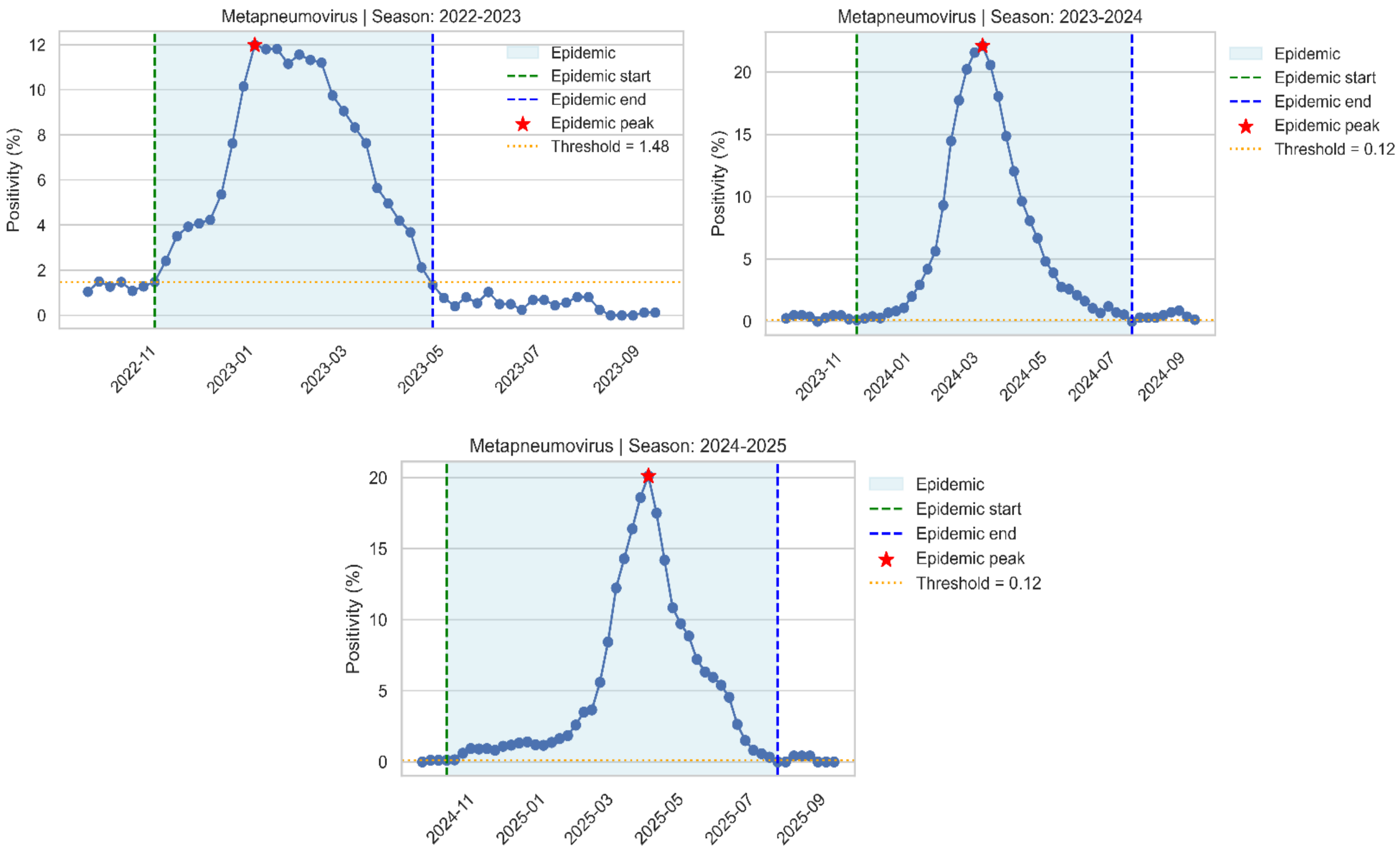

Figure S10. Definition of each epidemic of parainfluenza Type 2.

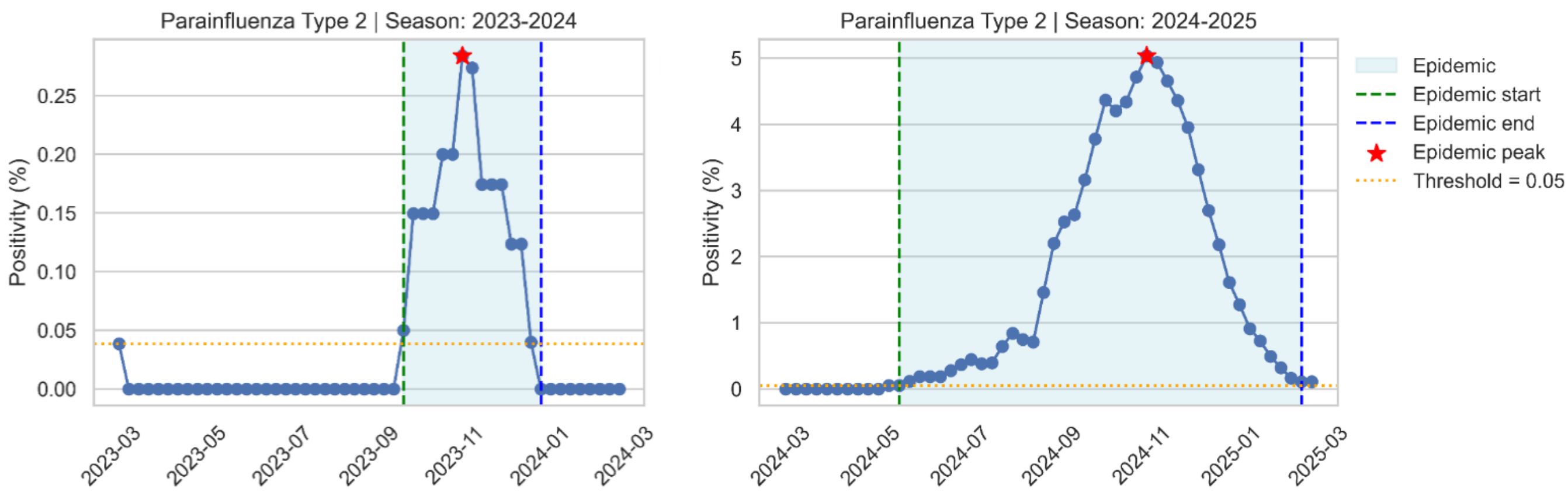

Figure S11. Definition of each epidemic of parainfluenza Type 3.

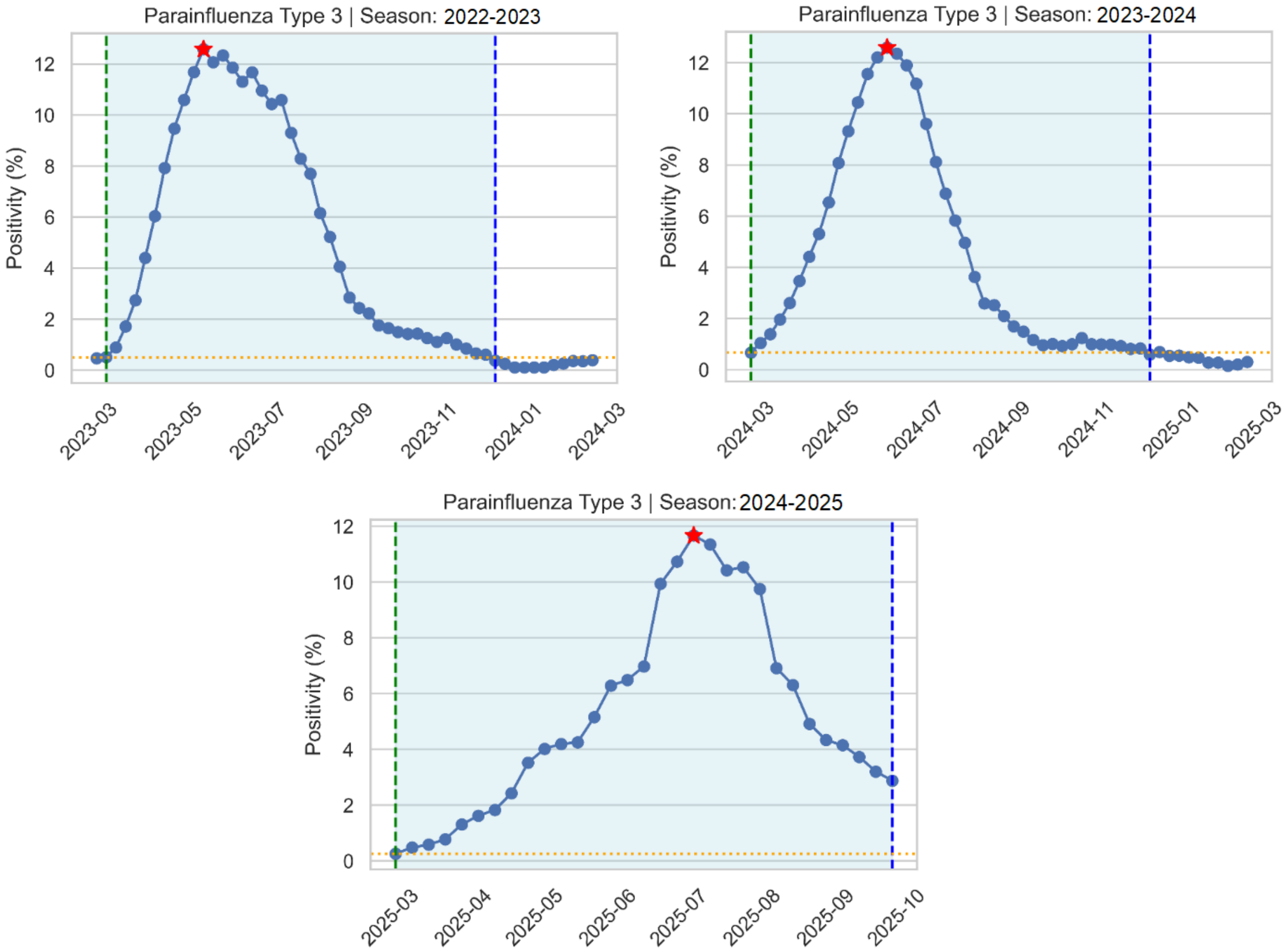

Figure S12. Definition of each epidemic of respiratory syncytial virus.

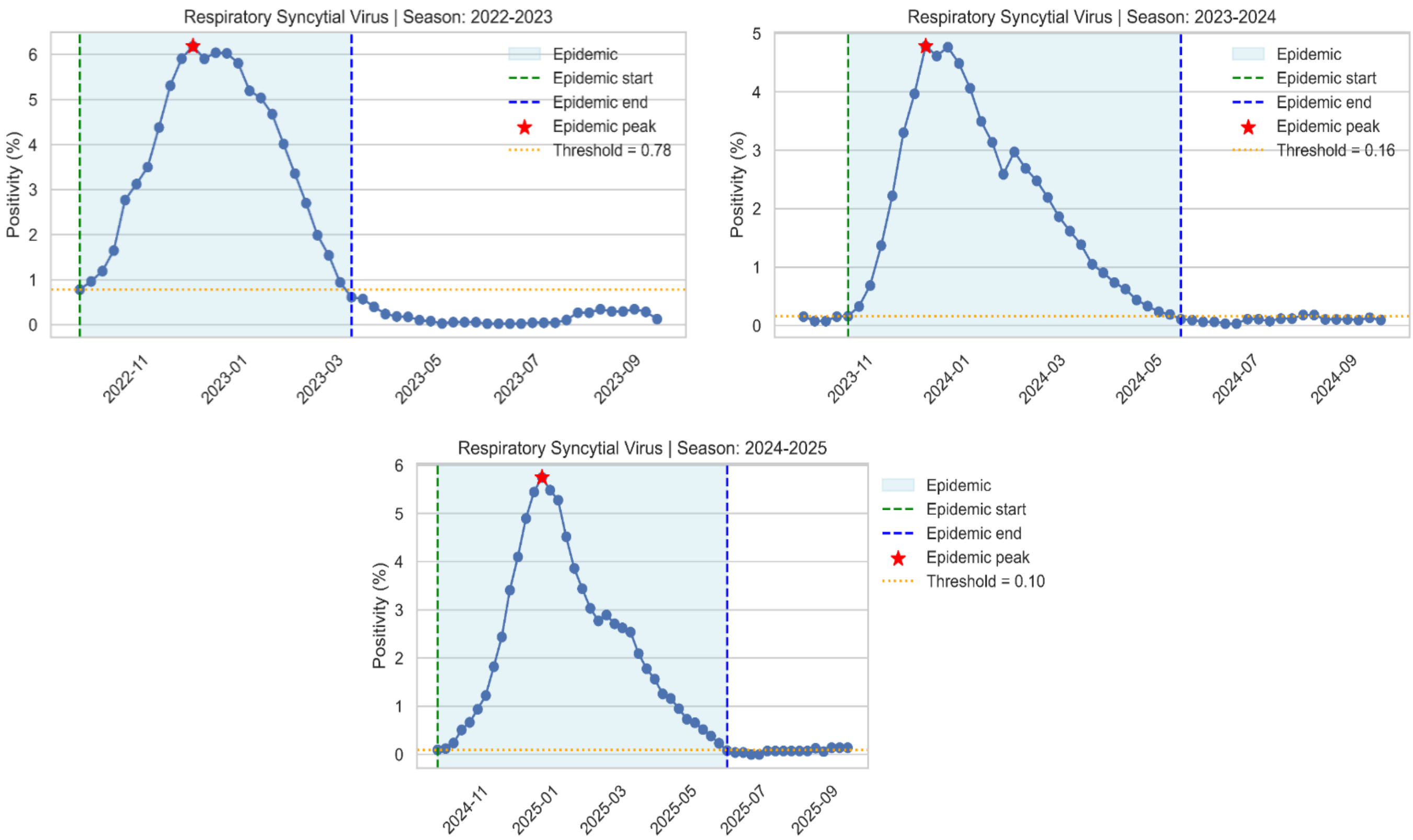

Figure S13. Definition of each epidemic of rhinovirus.

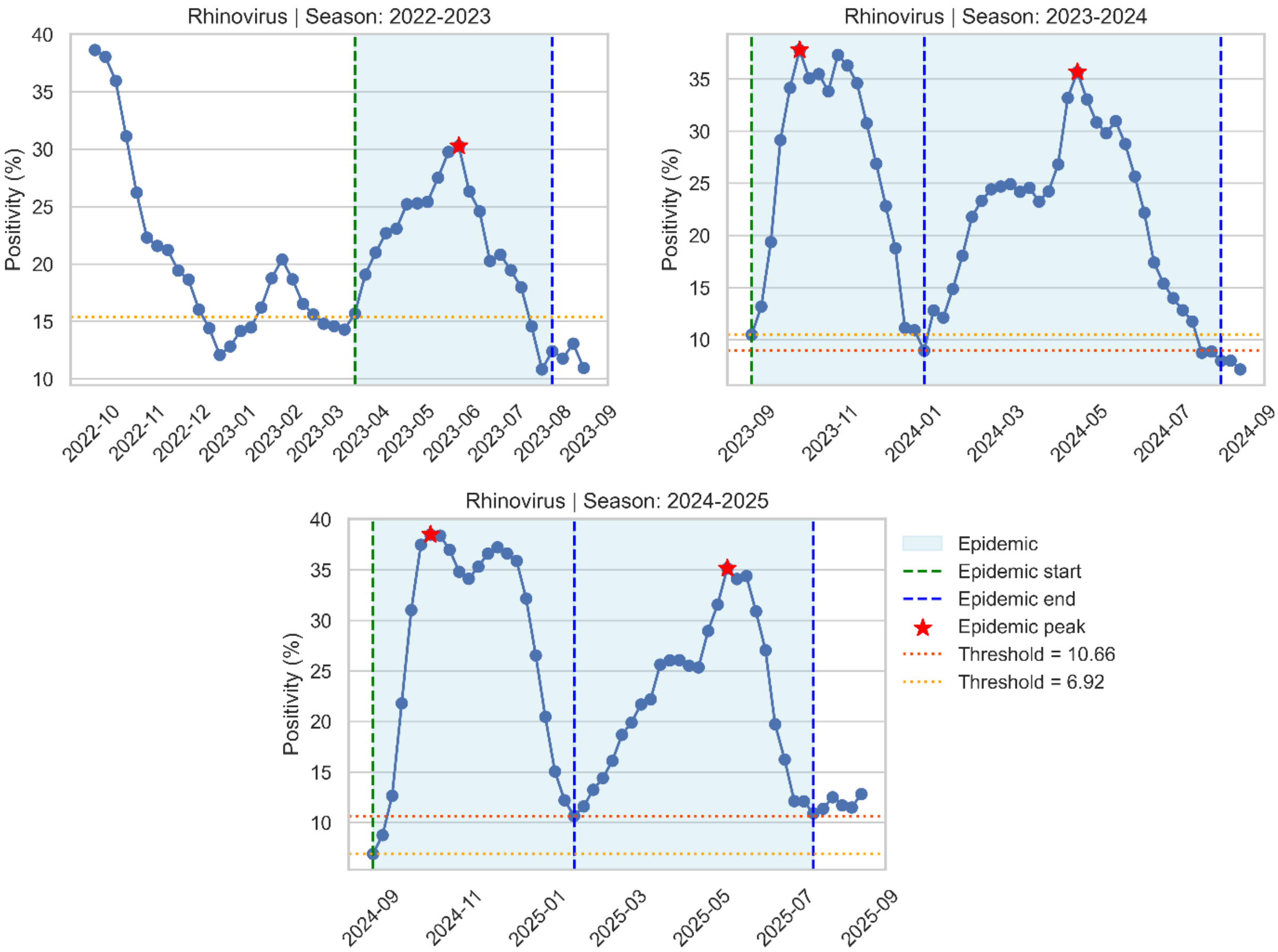

Figure S14. Definition of each epidemic of SARS-CoV-2. Notice that epidemic 2024-2025 is still incomplete.

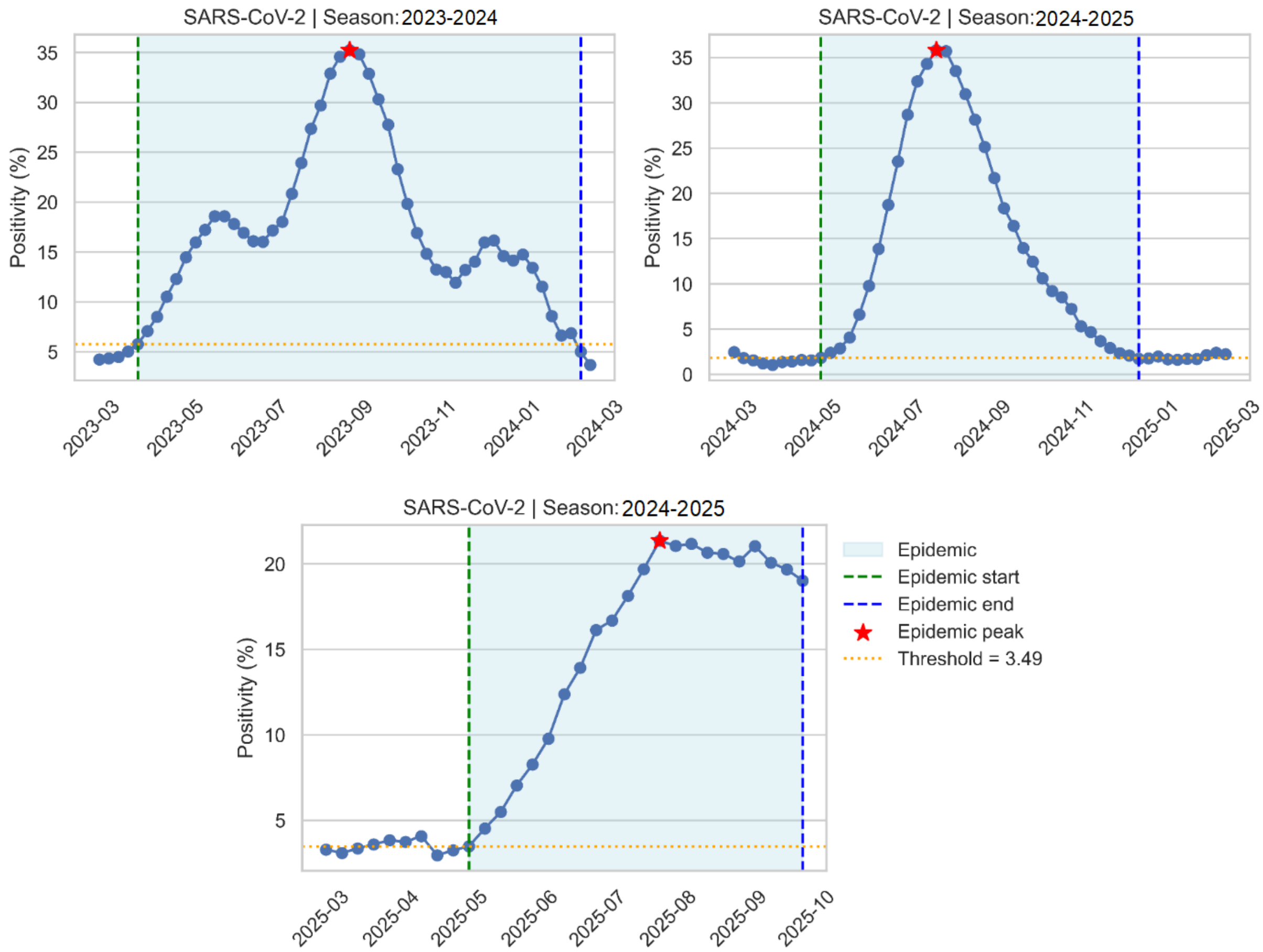

Figure S15. Delay between average epidemic starts, in weeks. Bottom viruses should be considered the reference to the week delay, therefore if the difference to a left virus is negative, this means that the left virus has a sooner epidemic start. Human rhinovirus and human enterovirus (1)/(2) refer to the first/second epidemic of their bimodal seasonality.

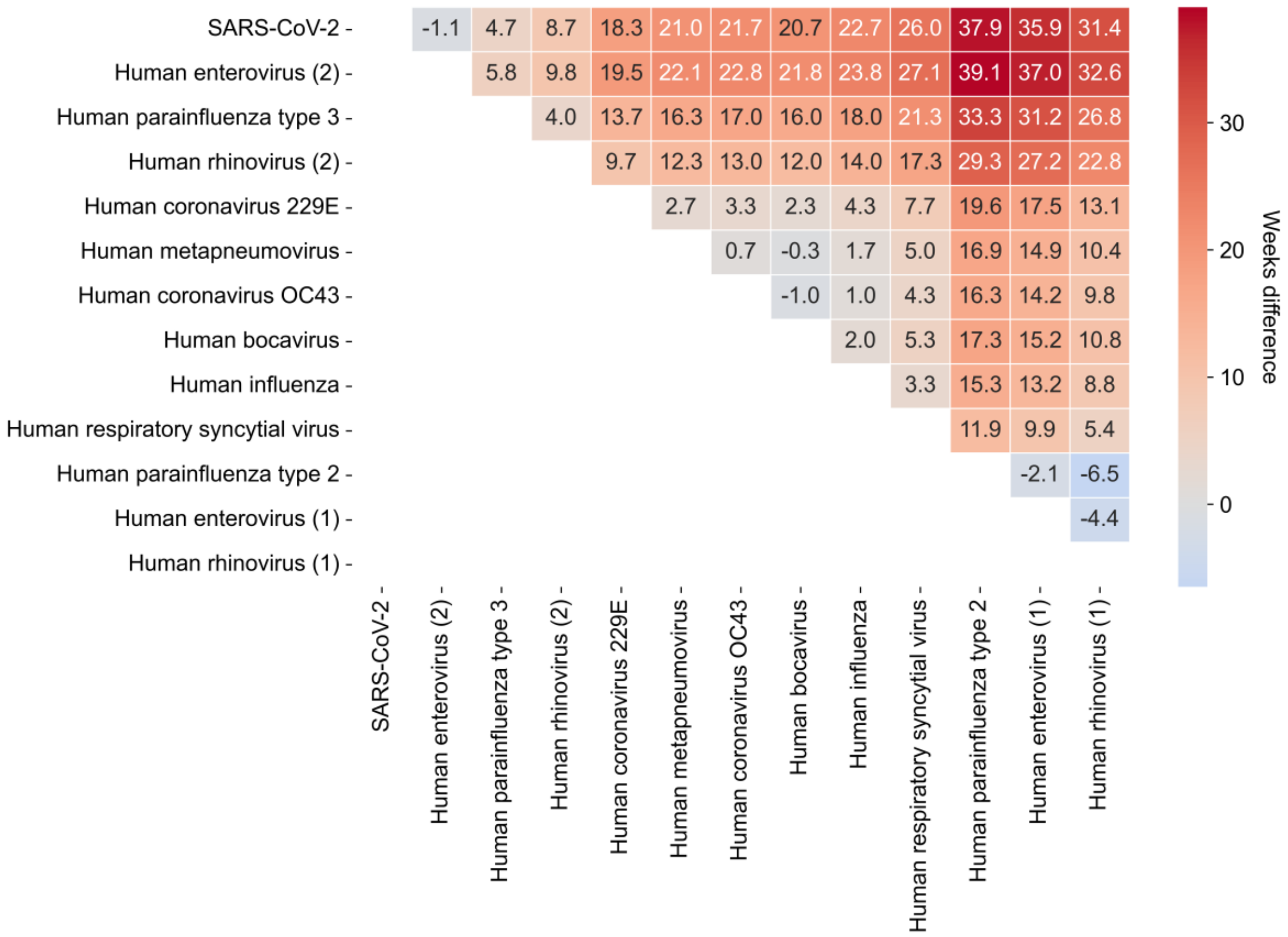

Figure S16. Delay between average epidemic peaks, in weeks. Bottom viruses should be considered the reference to the week delay, therefore if the difference to a left virus is negative, this means that the left virus has a sooner epidemic peak. Human rhinovirus and human enterovirus (1)/(2) refer to the first/second epidemic of their bimodal seasonality.

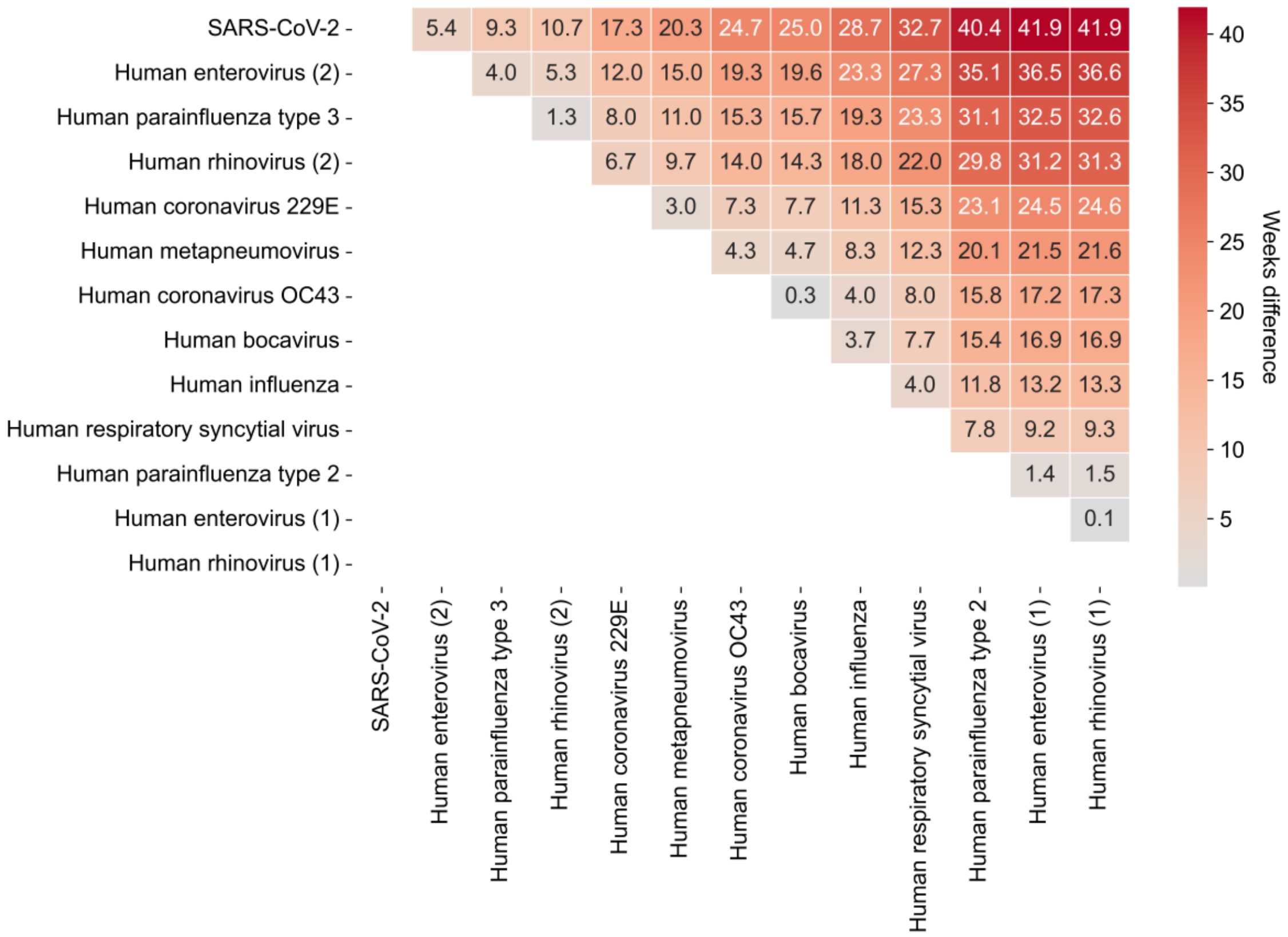

Figure S17. Delay between average epidemic ends, in weeks. Bottom viruses should be considered the reference to the week delay, therefore if the difference to a left virus is negative, this means that the left virus has a sooner epidemic end. Human rhinovirus and human enterovirus (1)/(2) refer to the first/second epidemic of their bimodal seasonality.

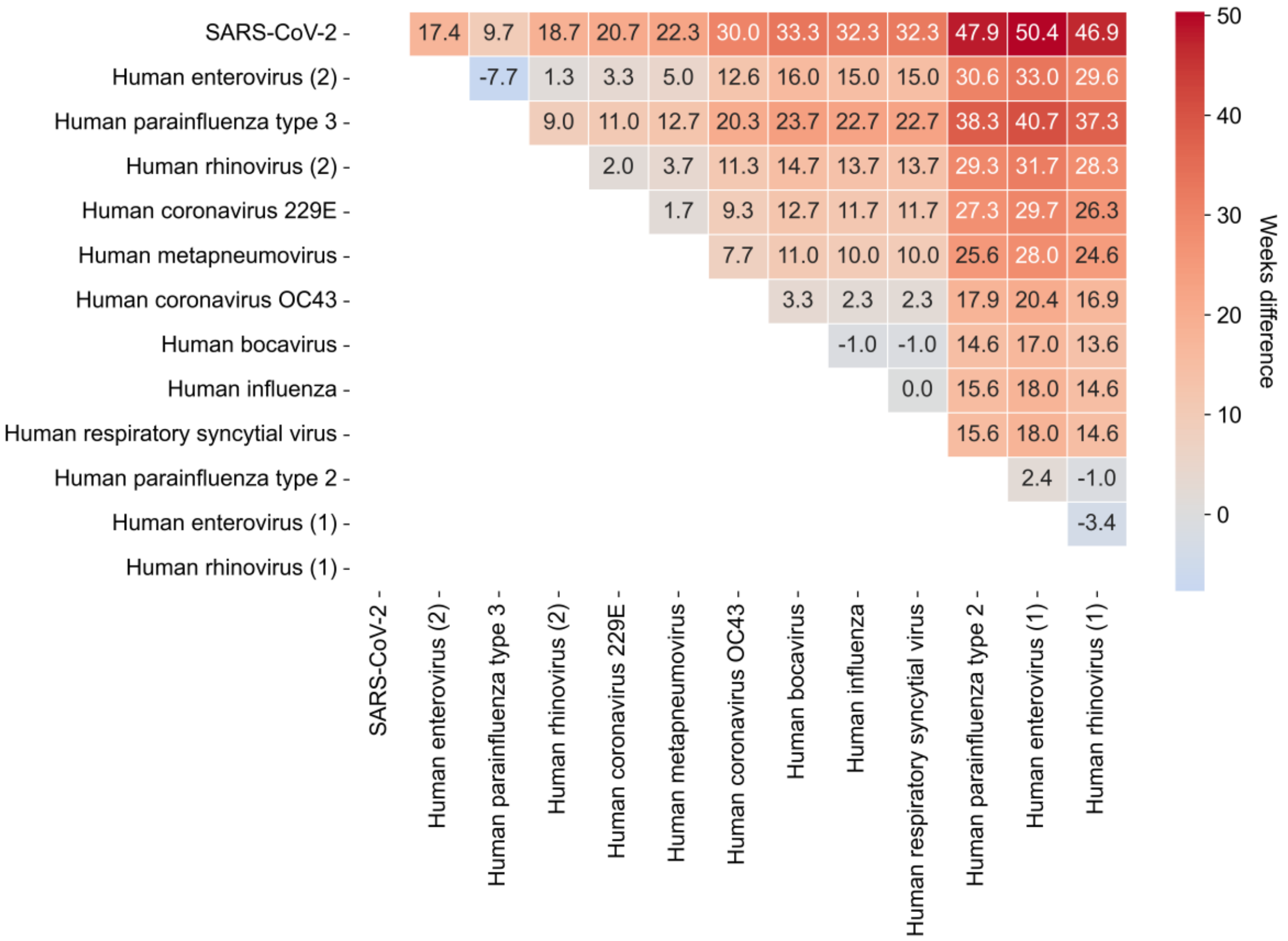

Supplement: Supplementary file 1 [file 26-00065_MENDIOROZ_Supplement.pdf]
